# The Efficacy of Mesenchymal Stem Cells in Therapy of Acute Kidney Injury Induced by Ischemia-Reperfusion in Animal Models

**DOI:** 10.1155/2020/1873921

**Published:** 2020-08-03

**Authors:** Tianbiao Zhou, Chunling Liao, Shujun Lin, Wenshan Lin, Hongzhen Zhong, Shuangyi Huang

**Affiliations:** Department of Nephrology, The Second Affiliated Hospital, Shantou University Medical College, 515041 Shantou, China

## Abstract

Mesenchymal stem cells (MSCs), discovered and isolated from the bone marrow in the 1960s and with self-renewal capacity and multilineage differentiation potential, have valuable immunomodulatory abilities. Acute kidney injury (AKI) refers to rapid renal failure, which exhibits as quickly progressive decreasing excretion in few hours or days. This study was performed to assess the efficacy of MSCs in the treatment of AKI induced by ischemia-reperfusion using a meta-analysis method. A literature search using corresponding terms was performed in the following databases: Embase, Cochrane Library, PubMed, and ISI Web of Science databases up to Dec 31, 2019. Data for outcomes were identified, and the efficacy of MSCs for AKI was assessed using Cochrane Review Manager Version 5.3. Nineteen studies were eligible and recruited for this meta-analysis. MSC treatment can reduce the Scr levels at 1 day, 2 days, 3 days, 5 days, and >7 days (1 day: WMD = −0.56, 95% CI: -0.78, -0.34, *P* < 0.00001; 2 days: WMD = −0.58, 95% CI: -0.89, -0.28, *P* = 0.0002; 3 days: WMD = −0.65, 95% CI: -0.84, -0.45, *P* < 0.00001; 5 days: WMD = −0.35, 95% CI: -0.54, -0.16, *P* = 0.0003; and >7 days: WMD = −0.22, 95% CI: -0.36, -0.08, *P* = 0.002) and can reduce the levels of BUN at 1 day, 2 days, 3 days, and 5 days (1 day: WMD = −11.72, 95% CI: -18.80, -4.64, *P* = 0.001; 2 days: WMD = −33.60, 95% CI: -40.15, -27.05, *P* < 0.00001; 3 days: WMD = −21.14, 95% CI: -26.15, -16.14, *P* < 0.00001; and 5 days: WMD = −8.88, 95% CI: -11.06, -6.69, *P* < 0.00001), and it also can reduce the levels of proteinuria at 3 days and >7 days and alleviate the renal damage in animal models of AKI. In conclusion, MSCs might be a promising therapeutic agent for AKI induced by ischemia-reperfusion.

## 1. Introduction

Mesenchymal stem cells (MSCs), discovered and isolated from bone marrow in the 1960s and with self-renewal capacity and multilineage differentiation potential, have valuable immunomodulatory abilities and exist in almost all human tissue lineages [1–3]. MSCs can secrete a wide range of growth factors, such as cytokines, chemokines, and extracellular vesicles—collectively termed the secretome [4, 5]. MSCs support revascularization, inhibition of inflammation, regulation of apoptosis, and promotion of the release of beneficial factors [6, 7]. MSC transplantation is a fast-developing therapy in cell-based therapies and regenerative medicine [8–10]. Thus, they are regarded as a promising candidate for the repair and regeneration of some diseases [6, 11–13].

Acute kidney injury (AKI) refers to rapid renal failure, which exhibits as quickly progressive decreasing excretion in few hours or days [14]. It is mainly characterized by oliguria or accumulation of serum creatinine, which is elevated by 0.3 mg/dl within 48 hours or more than 50% of the baseline [15, 16]. Ischemia-reperfusion is one of the common pathological conditions in AKI. It indicates that organs regain perfusion after temporary restriction of blood flow. In response to the sudden interruption of blood supply in IRI, oxidative stress and inflammation appear frequently in AKI [17, 18]. A series of cytokines, such as interleukins and tumor necrosis factor-*α* (TNF-*α*), are activated in this procedure. By promoting oxidative stress or apoptotic processes, they finally enhance renal inflammation and dysfunction [18–20].

This study was performed to assess the efficacy of MSCs in the treatment of AKI induced by ischemia-reperfusion using a meta-analysis method.

## 2. Materials and Methods

### 2.1. Search Strategy

A comprehensive search strategy for literature, which was restricted to English-language literature, was conducted in the Embase, Cochrane Library, PubMed, and ISI Web of Science databases up to Dec 31, 2019, using the following search corresponding terms: (mesenchymal stem cells OR MSC OR MSCs OR multipotent stromal cells OR mesenchymal stromal cells OR mesenchymal progenitor cells OR stem cells OR stromal cells) AND (acute kidney injury OR AKI OR acute renal failure OR ARF OR renal ischemia-reperfusion). The manual reference searches in the recruited articles were also conducted to identify additionally eligible reports.

### 2.2. Inclusion and Exclusion Criteria

#### 2.2.1. Inclusion Criteria

The inclusion criteria are the following: (1) research object: animal experiment used mice or rat, (2) object of the study: AKI, (3) interventions for study: MSCs for treatment, and (4) outcome: efficacy.

#### 2.2.2. Exclusion Criteria

The exclusion criteria are the following: (1) letters, case reports, reviews, clinical studies, editorials, meta-analysis, and systematic reviews; (2) studies lacking the targeted indicators or number of the case group or the control group and conducted in humans; (3) the AKI disease not induced by ischemia-reperfusion; and (4) the therapeutic regimen for AKI including other agents with undefined effects.

### 2.3. Outcome Measures

The following outcomes regarding the efficacy of MSC treatment on AKI induced by ischemia-reperfusion were identified from the recruited studies: serum creatinine (Scr), blood urea nitrogen (BUN), proteinuria, malondialdehyde (MDA), L-glutathione (GSH), CAT, superoxide dismutase (SOD), NADPH oxidase-1 (NOX1), NADPH oxidase-2 (NOX2), poly(ADP-ribose) polymerase-1 (PARP1), Caspase 3 (mRNA and protein), tumor necrosis factor-*α* (TNF-*α*), Bcl-2 associated X protein (Bax), nuclear factor kappa beta (NF*κ*B), interleukin 1*β* (IL1*β*; mRNA and protein), interleukin 4 (IL4), interleukin 6 (IL6) mRNA, interleukin 10 (IL10; mRNA and protein), transforming growth factor-*β*1 (TGF-*β*), and renal damage score. When disagreements were addressed, a mutual consensus was conducted to resolve it.

### 2.4. Quality Assessment

The Cochrane Handbook for Interventions was used to evaluate the methodological quality by two investigators independently (Tianbiao Zhou and Chunling Liao). The principal assessment included the following sections for each investigation: selection bias, attrition bias, performance bias, detection bias, reporting bias, and other bias. Each item was classified as unclear, high risk, or low risk.

### 2.5. Statistical Analysis

Review Manager Version 5.3 was used to explore whether MSC treatment can get a good efficacy on AKI induced by ischemia-reperfusion, and STATA 12.0 was applied to test the publication bias. Heterogeneity of variation among individual studies was quantified and described with *I*^2^. When the *P* value was ≥0.1, the fixed-effects model was used, based on the heterogeneity test. Otherwise, we will use the random-effects model to pool the results for the meta-analysis. Weighted mean differences (WMDs) for the mean values were used to compute the continuous variables, and 95% confidence intervals (95% CI) were calculated for the included studies using the Mantel-Haenszel (M-H) method. Both Begg's rank correlation test and Egger's linear regression method were applied to detect the publication bias among the studies. A *P* value < 0.05 was considered as statistical significance.

## 3. Results

### 3.1. Search Results

**Figure 1:**
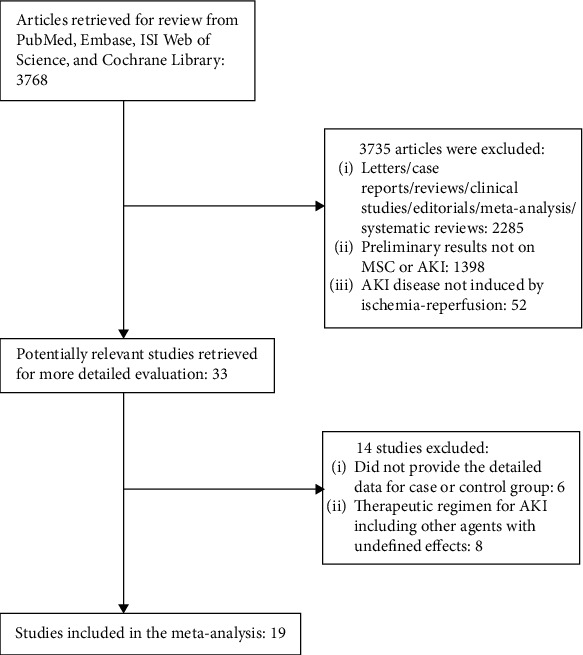
Flow diagram of the selection process.

The databases mentioned above were searched for this meta-analysis, and we only recruited these studies in mice or rat for evaluation of therapeutic efficiency of MSC treatment on AKI. Nineteen studies [21–39] were eligible and recruited for this meta-analysis, and the flowchart of inclusion of studies is presented in Figure 1. The included study characteristics are shown in Table 1.

### 3.2. Quality Assessment of Included Studies

**Figure 2:**
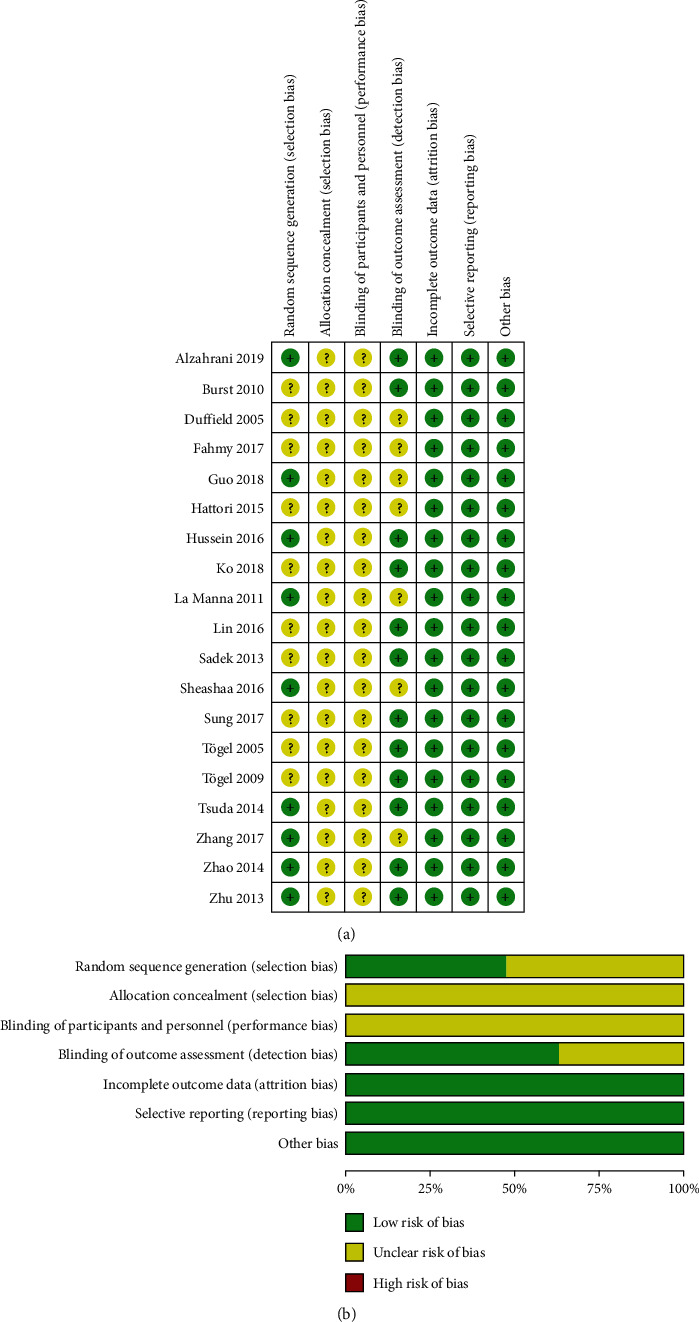
(a) Aggregate risk of bias graph for each experimental animal studies; (b) risk of bias summary.

In the recruited studies, the methodological quality was considered as acceptable, for the result that most of the domains of the recruited investigations were ranked as unclear risk of bias or low risk of bias. Unclear risk of bias was mostly detected in performance bias and selection bias. Low risk of bias mostly occurred in detection bias, reporting bias, and attrition bias.  Figure 2 shows the summary of the risk of biases of the recruited investigations.

### 3.3. Scr

**Figure 3:**
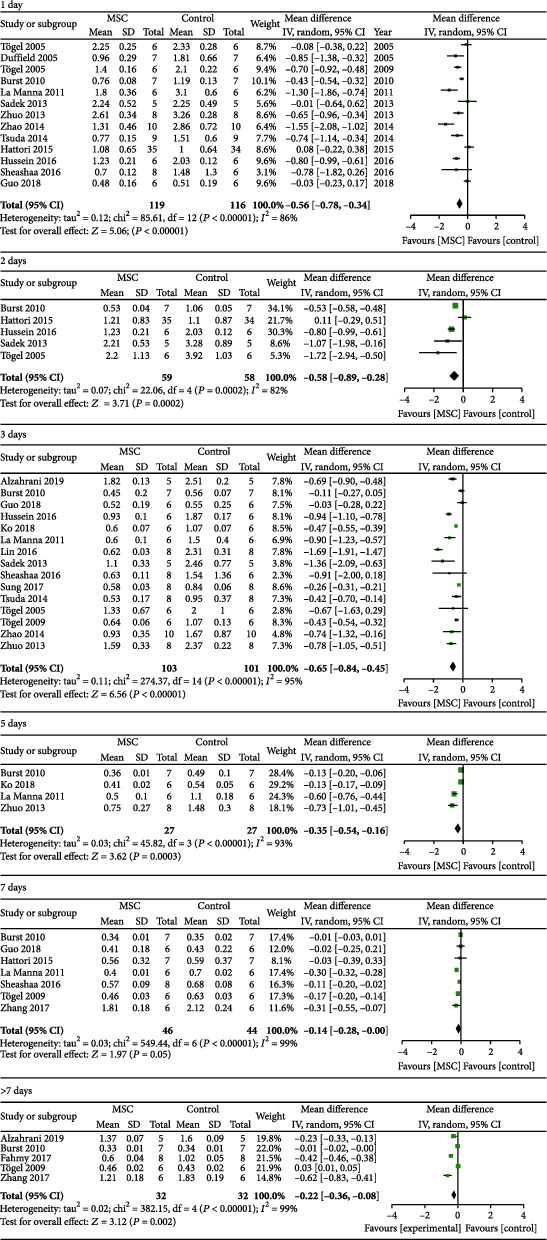
Effect of MSC on Scr.

19 studies [21–39] were included to assess the effect of MSCs on Scr, 12 for 1 day, four for 2 days, 14 for 3 days, four for 5 days, seven for 7 days, and five for >7 days, and the results showed that the difference between the MSC treatment group and the control group was notable for 1 day, 2 days, 3 days, 5 days, and >7 days (1 day: WMD = −0.56, 95% CI: -0.78, -0.34, *P* < 0.00001; 2 days: WMD = −0.58, 95% CI: -0.89, -0.28, *P* = 0.0002; 3 days: WMD = −0.65, 95% CI: -0.84, -0.45, *P* < 0.00001; 5 days: WMD = −0.35, 95% CI: -0.54, -0.16, *P* = 0.0003; and >7 days: WMD = −0.22, 95% CI: -0.36, -0.08, *P* = 0.002; Figure 3 and Table 2). However, the difference between the MSC treatment group and the control group was not notable for 7 days (WMD = −0.14, 95% CI: -0.28, -0.00, *P* = 0.05; Figure 3 and Table 2).

### 3.4. BUN

**Figure 4:**
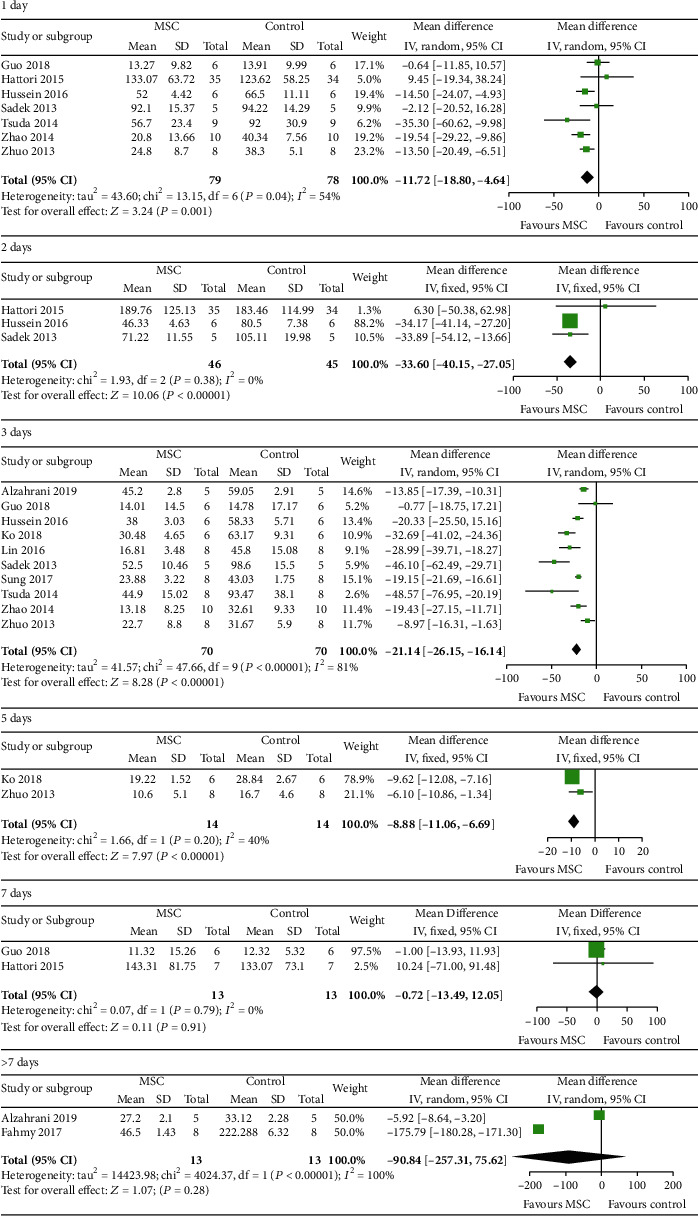
Effect of MSC on BUN.

12 studies [21, 24–28, 30, 31, 33, 36, 38, 39] were included to assess the effect of MSCs on Scr, 7 for 1 day, 3 for 2 days, 10 for 3 days, 2 for 5 days, 2 for 7 days, and 2 for >7 days, and the results indicated that the difference between the MSC treatment group and the control group was notable for 1 day, 2 days, 3 days, and 5 days (1 day: WMD = −11.72, 95% CI: -18.80, -4.64, *P* = 0.001; 2 days: WMD = −33.60, 95% CI: -40.15, -27.05, *P* < 0.00001; 3 days: WMD = −21.14, 95% CI: -26.15, -16.14, *P* < 0.00001; and 5 days: WMD = −8.88, 95% CI: -11.06, -6.69, *P* < 0.00001; Figure 4 and Table 2). However, the difference between the MSC treatment group and the control group was not notable for 7 days and >7 days (7 days: WMD = −0.72, 95% CI: -13.49, -12.05, *P* = 0.91; >7 days: WMD = −90.84, 95% CI: -257.31, 75.62, *P* = 0.28; Figure 4 and Table 2).

### 3.5. Proteinuria

Five studies [24, 28, 30, 33, 37] were recruited into the meta-analysis for the assessment of MSCs on proteinuria, three for 3 days and two for >7 days. The results showed that the MSC group had lower proteinuria than the control group for 3 days and for >7 days (3 days: WMD = −0.45, 95% CI: -0.61, -0.30, *P* < 0.00001; >7 days: OR = −108.55, 95% CI: -110.31, -106.78, *P* < 0.00001; Table 2).

### 3.6. Oxidative Stress and Apoptosis-Related Factors

In this meta-analysis, four studies [21, 24, 32, 39] were included for the assessment of MDA, two [24, 39] for GSH, two [21, 24] for CAT, two [21, 39] for SOD, three [28, 30, 33] for NOX1, four [21, 28, 30, 33] for NOX2, four [21, 28, 30, 33] for PARP1, two [21, 27] for Caspase 3 (mRNA), three [28, 30, 33] for Caspase 3 (protein), and three [28, 30, 33] for Bax. The results indicated that the difference between the MSC treatment group and the control group was notable for MDA, SOD, NOX1, NOX2, PARP1, Caspase 3 mRNA, Caspase 3 protein, and Bax (MDA: WMD = −5.51, 95% CI: -10.57, -0.45, *P* = 0.03; SOD: WMD = 18.95, 95% CI: 16.86, 21.04, *P* < 0.00001; NOX1: WMD = −0.32, 95% CI: -0.54, -0.10, *P* = 0.004; NOX2: WMD = −0.19, 95% CI: -0.28, -0.10, *P* < 0.0001; PARP1: WMD = −0.22, 95% CI: -0.34, -0.09, *P* = 0.0006; Caspase 3 mRNA: WMD = −3.40, 95% CI: -6.13, -0.68, *P* = 0.01; Caspase 3 protein: WMD = −0.15, 95% CI: -0.21, -0.08, *P* < 0.00001; and Bax: WMD = −0.25, 95% CI: -4.42, -0.08, *P* = 0.004; Table 2). However, the difference for GSH and CAT between the MSC treatment and the control group was not significant (GSH: WMD = −31.40, 95% CI: -21.52, 84.31, *P* = 0.24; CAT: WMD = 10.82, 95% CI: -4.30, 25.95, *P* = 0.16; Table 2).

### 3.7. Assessment of Cytokines

The levels of TNF-*α*, NF*κ*B, IL1*β* (mRNA), IL1*β* (protein), IL4, IL6 (mRNA), IL10 (mRNA), IL10 (protein), and TGF-*β* were detected, and five studies [25, 28, 30, 33, 37] for TNF-*α*, three studies [28, 30, 33] for NF*κ*B, two studies [21, 37] for IL1*β* (mRNA), three studies [25, 30, 33] for IL1*β* (protein), two studies [28, 33] for IL4, two studies [37, 38] for IL6 (mRNA), two studies [21, 37] for IL10 (mRNA), three studies [25, 28, 33] for IL10 (protein), and two studies [30, 37] for TGF-*β* were recruited for the evaluation of the treatment effect of MSC treatment on these cytokines. We also found that the difference between the MSC treatment group and the control group was significant for NF*κ*B, IL1*β* mRNA and protein, IL4, and IL10 mRNA and protein (NF*κ*B: WMD = −0.36, 95% CI: -0.66, -0.05, *P* = 0.02; IL1*β* mRNA: WMD = −3.26, 95% CI: -4.37, -2.15, *P* < 0.00001; IL1*β* protein: WMD = −0.37, 95% CI: -0.57, -0.17, *P* = 0.0003; IL4: WMD = 0.13, 95% CI: 0.02, 0.23, *P* = 0.02; IL10 mRNA: WMD = 0.27, 95% CI: 0.24, 0.29, *P* < 0.00001; and IL10 protein: WMD = 0.45, 95% CI: 0.04, 0.86, *P* = 0.03; Table 2). However, the difference for TNF-*α*, IL6 mRNA, and TGF-*β* between the MSC treatment and control groups was not significant (TNF-*α*: WMD = −0.15, 95% CI: -0.31, -0.02, *P* = 0.08; IL6 mRNA: WMD = −2.34, 95% CI: -4.75, 0.07, *P* = 0.06; and TGF-*β*: WMD = −18.89, 95% CI: -55.79, 18.02, *P* = 0.32; Table 2).

### 3.8. Assessment of Renal Damage Score

Four studies [29, 35, 36, 39] for 1 day and four studies [21, 30, 33, 36] for 3 days were included in this meta-analysis. The results indicated that the difference of the renal damage score for 1 day and for 3 days between the MSC treatment and control groups was significant (1 day: WMD = −14.50, 95% CI: -19.10, -9.90, *P* < 0.00001; 3 days: WMD = −1.19, 95% CI: -1.72, -0.66, *P* < 0.0001; Table 2).

### 3.9. Publication Bias

**Figure 5:**
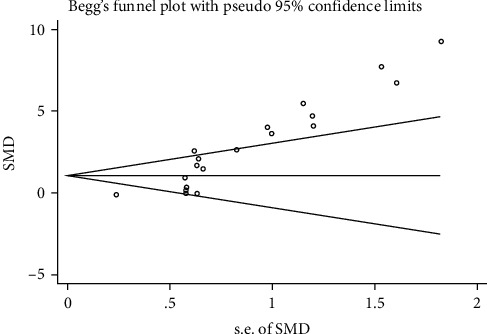
Publication bias.

The publication bias was tested in this meta-analysis, and a funnel plot was generated used STATA 12.0 for the primary outcome, and Begg's test and Egger's test suggested that publication bias was found (Egger's: *P* = 0.000, Begg's: *P* = 0.000; Figure 5).

## 4. Discussion

In this study, we found that MSC treatment can reduce the Scr levels at 1 day, 2 days, 3 days, 5 days, and >7 days in animal models of AKI. Furthermore, MSC treatment also can reduce the levels of BUN at 1 day, 2 days, 3 days, and 5 days, and it also can reduce the levels of proteinuria at 3 days and >7 days. The renal damage score was also detected, and we found that MSC treatment can significantly reduce the renal damage score in animal models of AKI. The results indicated that MSCs can get a protective role against AKI.

The dysfunction of oxidative stress is associated with AKI induced by ischemia-reperfusion, and cell injury or cell apoptosis takes part in the pathogenesis of AKI. As those mentioned above, the result indicated that the MSCs can improve the injury of AKI in animal models. We further collected the data about oxidative stress and apoptosis-related factors. In this study, the results indicated that MSC treatment can reduce MDA, NOX1, NOX2, PARP1, Caspase 3, and Bax and increase SOD. Previously, there were some studies indicating that MSC treatment can suppress oxidative stress and take the protective role. Song et al. [40] conducted a study in adriamycin-induced nephropathy rats and reported that MSCs can attenuate the nephropathy by diminishing oxidative stress and inhibiting the inflammation via downregulation of NF*κ*B. de Godoy et al. [41] evaluate the neuroprotective potential of MSCs against the deleterious impact of amyloid-*β* peptide on hippocampal neurons and reported that MSCs protect hippocampal neurons against oxidative stress and synapse damage. Chang et al. [42] reported that MSC transplantation successfully alleviates glomerulonephritis through antioxidation and antiapoptosis in nephritic rats.

Activation of some cytokines takes part in the pathogenesis of AKI induced by ischemia-reperfusion. In our study, we found that MSC treatment can inhibit NF*κ*B and IL1*β* and increased IL4 and IL10. Song et al. [40] indicated that MSCs can attenuate the nephropathy by inhibiting oxidative stress and alleviating the inflammation via inhibiting NF*κ*B. There were also some studies reporting the association of MSCs with ILs.

However, there were some limitations in our meta-analysis. First, the sample size for the recruited investigation was small, and the longer-term endpoints were missed. Furthermore, the animal type was different (mouse and rat), and the normal values of the parameters, such as BUN and Scr, for rats or mice were different. The type of MSCs and the dose of MSCs administered were not exactly the same. These factors mentioned above may cause our results to be less robust.

## 5. Conclusions

MSC treatment can reduce the Scr levels at 1 day, 2 days, 3 days, 5 days, and >7 days and can reduce the levels of BUN at 1 day, 2 days, 3 days, and 5 days, and it also can reduce the levels of proteinuria at 3 days and >7 days and alleviate the renal damage in animal models of AKI. The results indicated that MSCs can get a protective role against AKI. However, more well-designed studies with larger sample sizes and longer-term endpoints should be conducted to identify additional and robust outcomes in the future.

## Figures and Tables

**Table 1:** Characteristics of the studies included in this meta-analysis.

Author, year	*n*	Type of animal	MSC type	Number of MSC	Route of delivery	Endpoints for this meta-analysis
Tögel, 2005	12	Rat	BM-MSCs	0.1 × 10^6^	Artery	Scr
Duffield, 2005	14	Mice	BM-MSCs	0.5 × 10^6^	Intravenous	Scr
Tögel, 2009	36	Rat	BM-MSCs	2 × 10^6^	Artery	Scr
Burst, 2010	28	Rat	BM-MSCs	2 × 10^6^	Intravenous	Scr
La Manna, 2011	12	Rat	FM-MSCs	1 × 10^6^	Intravenous	Scr, renal damage score
Zhuo, 2013	24	Rat	BM-MSCs	1 × 10^6^	Intravenous or artery	Scr, BUN, MDA, GSH, SOD, renal damage score
Sadek, 2013	10	Rat	BM-MSCs	—	Intravenous	Scr, BUN
Zhao, 2014	20	Rat	BM-MSCs	1 × 10^6^	Intravenous	Scr, BUN, IL6 mRNA
Tsuda, 2014	54	Rat	FM-MSCs	0.5 × 10^6^	Intravenous	Scr, BUN, renal damage score
Hattori, 2015	22	Mice	BM-MSCs	1 × 10^6^	Kidney subcapsular injection	Scr, BUN
Lin, 2016	16	Rat	AD-MSCs	1.2 × 10^6^	Intravenous	Scr, BUN, proteinuria, NOX1, NOX2, PARP1, Caspase 3 protein, Bax, TNF-*α*, NF*κ*B, IL1*β* protein, TGF-*β*, renal damage score
Hussein, 2016	36	Rat	AD-MSCs	1 × 10^6^	Intravenous	Scr, BUN, Caspase 3 mRNA
Sheashaa, 2016	42	Rat	AD-MSCs	1 × 10^6^	Intravenous	Scr, MDA
Zhang, 2017	12	Rat	AD-MSCs	2 × 10^6^	Intravenous	Scr, proteinuria, TNF-*α*, IL1*β* mRNA, IL6 mRNA, IL10 mRNA, TGF-*β*
Fahmy, 2017	16	Rat	UC-MSCs	1 × 10^6^	Intravenous	Scr, BUN, proteinuria, MDA, GSH, CAT
Sung, 2017	16	Rat	AD-MSCs	1.2 × 10^6^	Intravenous	Scr, BUN, proteinuria, NOX1, NOX2, PARP1, Caspase 3 protein, Bax, TNF-*α*, NF*κ*B, IL1*β* protein, IL4, IL10 protein, renal damage score
Guo, 2018	36	Mice	UC-MSCs	1 × 10^6^	Intravenous	Scr, BUN, TNF-*α*, IL1*β* protein, IL10 protein
Ko, 2018	12	Rat	iPSC-MSC	1.2 × 10^6^	Intravenous	Scr, BUN, proteinuria, NOX1, NOX2, PARP1, Caspase 3 protein, Bax, TNF-*α*, NF*κ*B, IL4, IL10 protein
Alzahrani, 2019	20	Rat	BM-MSCs	1 × 10^6^	Artery	Scr, BUN, MDA, CAT, SOD, NOX2, PARP1, Caspase 3 mRNA, IL1*β* mRNA, IL10 mRNA, renal damage score

Note: FM-MSCs: fetal membrane-derived mesenchymal stem cells; BM-MSC: bone marrow-derived mesenchymal stem cells; AD-MSCs: adipose tissue-derived MSCs; UC-MSCs: umbilical cord mesenchymal stem cells; iPSC-MSCs: inducible pluripotent stem cell-derived mesenchymal stem cells; Scr: serum creatinine; BUN: blood urea nitrogen; MDA: malondialdehyde; GSH: L-glutathione; SOD: superoxide dismutase; NOX1: NADPH oxidase-1; NOX2: NADPH oxidase-2; PARP1: poly(ADP-ribose) polymerase-1; TNF-*α*: tumor necrosis factor-*α*; Bax: Bcl-2 associated X protein; NF*κ*B: nuclear factor kappa beta; IL1*β*: interleukin 1*β*; IL4: interleukin 4; IL6: interleukin 6; IL10: interleukin 10; TGF-*β*: transforming growth factor-*β*.

**Table 2:** Meta-analysis of the efficacy of MSC in therapy of acute kidney injury induced by ischemia-reperfusion.

Indicators	Timepoint	Study number	*Q* test *P* value	Model selected	WMD (95% CI)	*P*
Scr	1 day	13	<0.00001	Random	-0.56 (-0.78, -0.34)	<0.00001
	2 days	5	0.0002	Random	-0.58 (-0.89, -0.28)	0.0002
	3 days	15	<0.00001	Random	-0.65 (-0.84, -0.45)	<0.00001
	5 days	4	<0.00001	Random	-0.35 (-0.54, -0.16)	0.0003
	7 days	7	<0.00001	Random	-0.14 (-0.28, -0.00)	0.05
	>7 days	5	<0.00001	Random	-0.22 (-0.36, -0.08)	0.002
BUN	1 day	7	0.04	Random	-11.72 (-18.80, -4.64)	0.001
	2 days	3	0.38	Fixed	-33.60 (-40.15, -27.05)	<0.00001
	3 days	10	<0.00001	Random	-21.14 (-26.15, -16.14)	<0.00001
	5 days	2	0.20	Fixed	-8.88 (-11.06, -6.69)	<0.00001
	7 days	2	0.79	Fixed	-0.72 (-13.49, -12.05)	0.91
	>7 days	2	<0.00001	Random	-90.84 (-257.31, 75.62)	0.28
Proteinuria	3 days	3	<0.00001	Random	-0.45 (-0.61, -0.30)	<0.00001
	>7 days	2	0.21	Fixed	-108.55 (-110.31, -106.78)	<0.00001
MDA	—	4	0.0001	Random	-5.51 (-10.57, -0.45)	0.03
GSH	—	2	0.0002	Random	-31.40 (-21.52, 84.31)	0.24
CAT	—	2	<0.00001	Random	10.82 (-4.30, 25.95)	0.16
SOD	—	2	0.41	Fixed	18.95 (16.86, 21.04)	<0.00001
NOX1	—	3	<0.00001	Random	-0.32 (-0.54, -0.10)	0.004
NOX2	—	4	<0.00001	Random	-0.19 (-0.28, -0.10)	<0.0001
PARP1	—	4	<0.00001	Random	-0.22 (-0.34, -0.09)	0.0006
Caspase 3 (mRNA)	—	2	<0.00001	Random	-3.40 (-6.13, -0.68)	0.01
Caspase 3 (protein)	—	3	<0.00001	Random	-0.15 (-0.21, -0.08)	<0.00001
Bax	—	3	<0.00001	Random	-0.25 (-4.42, -0.08)	0.004
TNF-*α*	—	5	<0.00001	Random	-0.15 (-0.31, -0.02)	0.08
NF*κ*B	—	3	<0.00001	Random	-0.36 (-0.66, -0.05)	0.02
IL1*β* (mRNA)	—	2	0.007	Random	-3.26 (-4.37, -2.15)	<0.00001
IL1*β* (protein)	—	3	<0.00001	Random	-0.37 (-0.57, -0.17)	0.0003
IL4	—	2	<0.00001	Random	0.13 (0.02, 0.23)	0.02
IL6 (mRNA)	—	2	<0.00001	Random	-2.34 (-4.75, 0.07)	0.06
IL10 (mRNA)	—	2	0.13	Fixed	0.27 (0.24, 0.29)	<0.00001
IL10 (protein)	—	3	<0.00001	Random	0.45 (0.04, 0.86)	0.03
TGF-*β*	—	2	<0.00001	Random	-18.89 (-55.79, 18.02)	0.32
Renal damage score	1 day	4	<0.00001	Random	-14.50 (-19.10, -9.90)	<0.00001
	3 days	4	<0.00001	Random	-1.19 (-1.72, -0.66)	<0.0001

Note: Scr: serum creatinine; BUN: blood urea nitrogen; MDA: malondialdehyde; GSH: L-glutathione; SOD: superoxide dismutase; NOX1: NADPH oxidase-1; NOX2: NADPH oxidase-2; PARP1: poly(ADP-ribose) polymerase-1; TNF-*α*: tumor necrosis factor-*α*; Bax: Bcl-2 associated X protein; NF*κ*B: nuclear factor kappa beta; IL1*β*: interleukin 1*β*; IL4: interleukin 4; IL6: interleukin 6; IL10: interleukin 10; TGF-*β*: transforming growth factor-*β*.

## Data Availability

The data supporting this meta-analysis are from previously reported studies and datasets, which have been cited. The processed data are available from the corresponding author upon request.
